# CD226 ligation protects against EAE by promoting IL-10 expression *via* regulation of CD4^+^ T cell differentiation

**DOI:** 10.18632/oncotarget.7834

**Published:** 2016-03-01

**Authors:** Rong Zhang, Hanyu Zeng, Yun Zhang, Kun Chen, Chunmei Zhang, Chaojun Song, Liang Fang, Zhuwei Xu, Kun Yang, Boquan Jin, Qintao Wang, Lihua Chen

**Affiliations:** ^1^ Department of Immunology, The Fourth Military Medical University, Xi'an, Shaanxi, P.R. China; ^2^ State Key Laboratory of Military Stomatology, Department of Periodontology, School of Stomatology, The Fourth Military Medical University, Xi'an, Shaanxi, P.R. China; ^3^ Department of Neurobiology, The Fourth Military Medical University, Xi'an, Shaanxi, P.R. China

**Keywords:** CD226, CD4^+^ T cells, IL-10, EAE, iTreg, Immunology and Microbiology Section, Immune response, Immunity

## Abstract

Treatment targeting CD226 can ameliorate experimental autoimmune encephalomyelitis (EAE), the widely accepted model of MS. However, the mechanisms still need to be elucidated. Here we showed that CD226 blockage by anti-CD226 blocking mAb LeoA1 efficiently promoted IL-10 production in human peripheral blood monocytes (PBMC) or in mixed lymphocyte culture (MLC) system, significantly induced the CD4^+^IL-10^+^ T cell differentiation while suppressing the generation of Th1 and Th17. Furthermore, CD226 pAb administration *in vivo* reduced the onset of EAE in mice by promoting IL-10 production and regulating T cell differentiation. Concomitantly, the onset and severity of EAE were reduced and the serum IL-10 expression levels were increased in CD226 knockout mice than that in control mice when both received EAE induction. These novel findings confirmed that CD226 played a pivotal role in mediating autoimmune diseases such as EAE. Furthermore, to our knowledge, we show for the first time that IL-10 is an important contributor in the inhibitory effects of CD226 ligation on EAE.

## INTRODUCTION

CD4^+^ T lymphocytes play an important role in regulating host immune responses as well as inflammatory and autoimmune diseases. As an important influencing factor, Th subsets differentiation disorders have been shown to be highly associated with the pathogenesis and progression of EAE [1]. Specifically, the proinflammatory IFN-γ-producing Th1 cells and IL-17 producing Th17 cells could be used in EAE induction, with different pathological phenotypes [1, 2]. Moreover, immune suppressive agent dihydroartemisinin was proved to be effective in ameliorating EAE by suppressing Th cell function, particularly by abolishing Th17 differentiation [3]. Interleukin-10 (IL-10), a cytokine with anti-inflammatory properties, plays a crucial role in preventing various inflammatory pathologies especially in tumor and autoimmune diseases [4, 5]. Administration of IL-10 has been demonstrated to suppress EAE under some conditions [6, 7]. Also, high IL-10 expression level within the central nervous system is considered to be important for the initiation of recovery from EAE [8]. The mechanism may lie in the inhibition of T cell subsets maturation and autoreactive CD4^+^ T cell expansion in the central nervous system (CNS). Meanwhile, IL-10-deficient mice develop more severe EAE than wild-type (WT) mice [9], thus demonstrating its beneficial effects on the pathogenesis of EAE.

CD226, also known as DNAX accessory molecule-1 (DNAM-1) or platelet and T cell activation antigen 1 (PTA1), is involved in various pathological processes including autoimmune diseases, tumor, transplantation rejection and virus infection diseases [10, 11]. As for the relationship between CD226 and EAE or MS, it was reported that anti-CD226 treatment delayed the onset and reduced the severity of Th1-mediated EAE [12]. Moreover, neutralizing anti-CD226 mAb decreased T cell activation and inhibited proliferation of CD4^+^ T cell isolated from untreated patients with relapsing remitting MS [13]. However, the mechanisms underlying how CD226 exerts its impact on EAE still needs to be further elucidated.

IL-10 is an important mediator which exerts multiple immunosuppressive actions, including modulation of APCs [14], inhibition of T cell proliferation [15], and maintaining the function/stability of established Tregs [16, 17]. Our previous studies in nearly 2000 showed that CD226 blocking mAb LeoA1 could efficiently promote the production of IL-10 in human PBMC and MLC culture systems (data not published). Given the important role of Th subsets and IL-10 in EAE, we investigated whether anti-CD226 Ab could ameliorate EAE *via* the alteration of IL-10 expression levels and the differentiation of Th subsets.

## RESULTS

### CD226 ligation promotes IL-10 production in human PBMC and MLC culture supernatants

**Figure 1:**
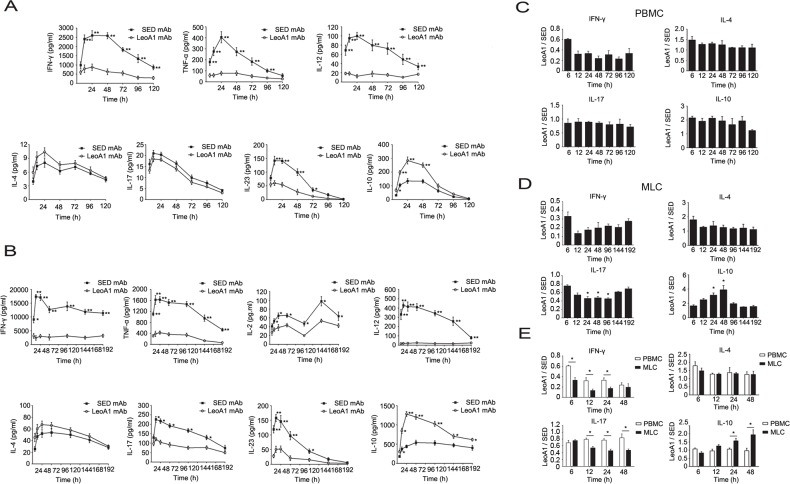
CD226 mAb LeoA1 upregulates IL-10 production in human PBMC and MLC culture supernatants **A.** Human PBMC were isolated from peripheral blood and cultured under the treatment of LeoA1 and SED mAb (negative control) for the indicated periods of time. The production of CD4^+^ T cell subsets associated cytokines in the supernatants were assessed by ELISA. **B.** The same experiments were repeated under the MLC system containing Daudi and PBMC as the stimulator cells and responding cells respectively for the indicated time. The ratio between the typical CD4^+^ T cells associated cytokines production of the two groups obtained from experiments described in (A and B) was calculated in PBMC **C.** and MLC **D.** systems respectively. *P < 0.05 by comparison to all the other bars without *. E. As described in (C and D), the ratio was compared between the PBMC and MLC systems at the four coincident time points. Data are representative of at least three independent experiments. Error bars denote SEM (A and B) or SD (C-E). *P < 0.05. **P < 0.01.

To assess the effect of the CD226 ligation on the cytokine secretion profile in human PBMC and MLC systems, we measured the IFN-γ, TNF-α, IL-12, IL-17, IL-23, IL-10, IL-2 and IL-4 expression levels in the supernatants of PBMC and MLC systems at different time points. We found that CD226 mAb LeoA1 decreased IFN-γ, TNF-α, IL-12 and IL-23 but increased IL-10 secretion in both systems and only decreased IL-2 and IL-17 expression levels in MLC system. However, the production of IL-4, which is predominantly secreted by Th2, almost remained the same in both systems (Figure 1A and 1B).

Then we analyzed whether the regulatory effect of LeoA1 was related to time course. By making a ratio of four representative cytokine expression levels between the LeoA1 and SED groups, we found that LeoA1 exerted the regulatory function to a stable extent without being affected by time in the PBMC system (Figure 1C). However, the expression levels of IL-17 and IL-10, referring to the MLC system, were altered much more significantly from 24h to 48h (Figure 1D). Furthermore, when the two culture systems were compared, LeoA1 performed a much fiercer effect on the three cytokine production (IFN-γ, IL-17, IL-10) in the MLC system and the most obvious elevated IL-10 expression level in MLC compared with that in PBMC was at 48h after the treatment. (Figure 1E).

### CD226 ligation up-regulates the frequencies of CD4^+^IL-10^+^ T cells in human PBMC and MLC culture systems

**Figure 2:**
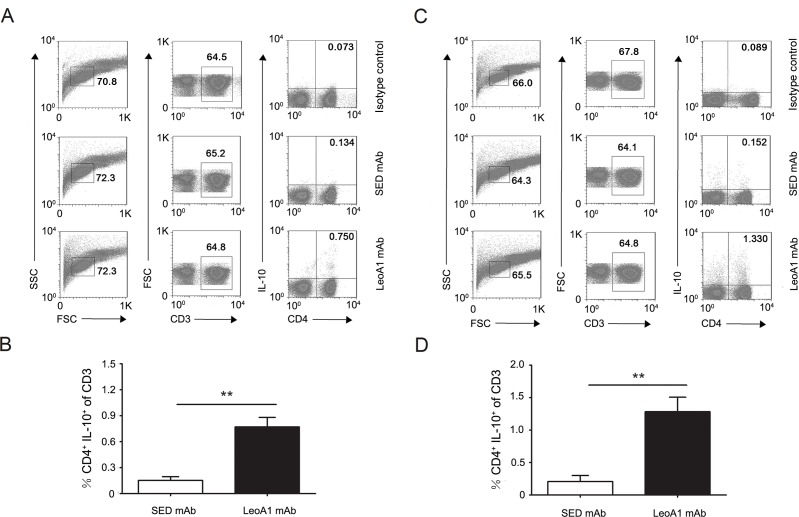
CD226 mAb LeoA1 promotes the differentiation of CD4^+^ IL-10^+^ T cells **A.** Human PBMC were cultured with LeoA1 or SED mAb for 24h and submitted to flow cytometry analysis by gating on CD3^+^ followed by surface expression of CD4 *versus* IL-10 and isotype control antibody intracellular staining on cells, stimulated with PMA and ionomycin for 4h in the presence of GolgiStop. Cells were stained with LIVE/DEAD Fixable Dead Cell Stain Kit before fixation to allow gating on viable cells. **B.** The frequencies of CD4^+^ IL-10^+^ in the total T cells from experiments described in (A) were compared. **C.** The same experiment as in (A) was duplicated under MLC system. **D.** The frequencies of CD4^+^ IL-10^+^ in the total T cells from experiments described in (C) were compared. Data are representative of at least three independent experiments. Error bars denote SD. **P < 0.01.

Considering the above results that CD226 ligation could significantly up-regulate IL-10 expression levels and IL-10 plays a crucial role in preventing inflammatory and autoimmune pathologies, we next explored whether CD226 mAb could promote the differentiation of IL-10^+^ immunocytes in PBMC. Flow cytometry analysis showed that in MLC system (Daudi as APC), after 24h treatment, LeoA1 had no obvious effect on IL-10^+^ proportion of DCs, macrophages, NK cells and B cells (Supplemental Figure 1), which can produce different amount of IL-10 [18]. However, the frequencies of IL-10 secreting CD4^+^ T cells were efficiently elevated from 0.134% to 0.750% and from 0.152% to 1.330% after LeoA1 treatment in PBMC (Figure 2A and 2B) and MLC (Figure 2C and 2D) system respectively. These data suggested that CD226 ligation promoted CD4^+^ IL-10^+^ T cell differentiation.

### CD226 ligation inhibits the production of Th1/Th17 associated genes and cytokines while promoting the iTreg associated ones

**Figure 3:**
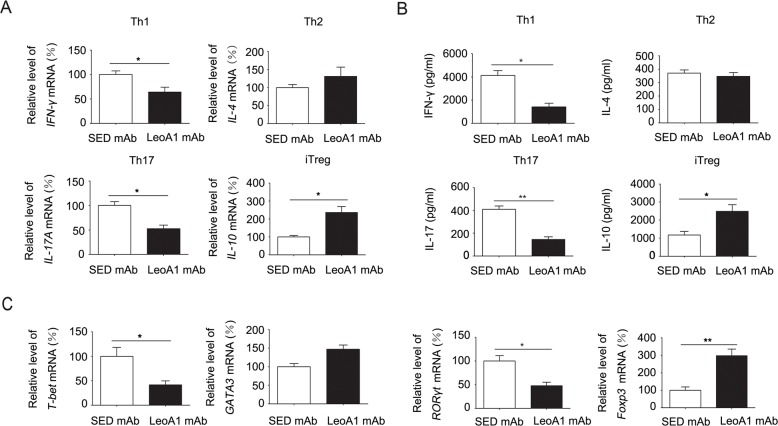
CD226 mAb LeoA1 inhibits the production of Th1/Th17 related genes and cytokines while promotes iTreg associated ones in human CD4^+^ T cells **A.** MACS-sorted CD4^+^ T cells from human PBMC were activated with anti-CD3 and anti-CD28 in the presence of LeoA1 or SED mAb under Th1, Th2, Th17 or iTreg conditions respectively for 4d. mRNA expression of IFN-γ, IL-4, IL-17 and IL-10 were analyzed by quantitative real-time RT-PCR. **B.** Measurement of IFN-γ, IL-4, IL-17 and IL-10 in the supernatants derived from the same cultures as in (A) was quantified by ELISA. **C.** The gene expression of Th1, Th2, Th17, iTreg associated master transcription factors in human CD4^+^ T cells were analyzed by quantitative real-time RT-PCR undergoing the same treatment as in (A). Data are representative of at least three independent experiments. Error bars denote SEM. *P < 0.05, **P < 0.01.

Considering the complexity of PBMC and MLC systems, to address the functional role of CD226 in cytokine production of CD4^+^ T cells, we purified human CD4^+^ T cells which were activated with anti-CD3 and anti-CD28, then with anti-CD226 or SED mAb under Th1, Th2, Th17 or iTreg conditions for 4d. As expected, anti-CD226 mAb LeoA1 inhibited the mRNA expression of IFN-γ and IL-17 while elevated the mRNA level of IL-10 (Figure 3A). Meanwhile, IFN-γ and IL-17 secretion levels were significantly decreased while IL-10 expression level was obviously elevated in the LeoA1 treatment group (Figure 3B). However, there is little difference on Th2 associated cytokine IL-4 expression on both mRNA and protein levels (Figure 3A and 3B). Furthermore, LeoA1 treated cells expressed lower levels of T-bet and RORγt (the master transcription factors for Th1 and Th17 respectively), higher level of Foxp3 (the master transcription factor for iTreg) and an unchanged level of GATA3 (key transcription factor for Th2) (Figure 3C). Collectively, our data strongly suggested that CD226 promoted iTreg differentiation while inhibited Th1/Th17 generation.

### CD226 pAb treatment *in vivo* reduces EAE susceptibility and severity

**Figure 4:**
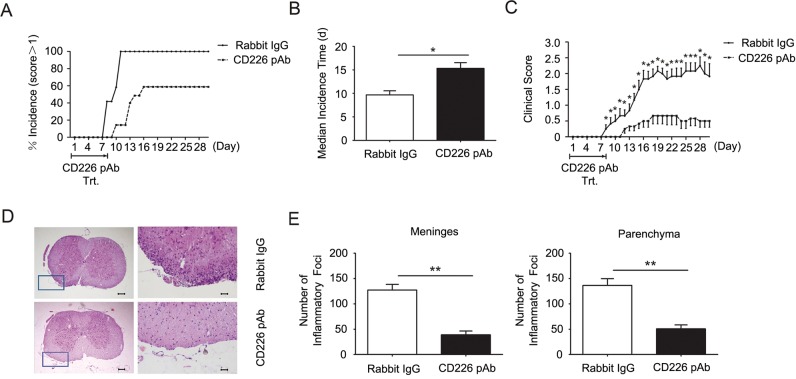
CD226 pAb treatment *in vivo* reduces EAE susceptibility and severity **A.** EAE was elicited in mice with CFA-MOG injection. Mice were administrated with rabbit anti-mouse CD226 pAb (dashed line) or rabbit IgG (solid line) every other day during the first 8 d of EAE elicitation. The numbers of mice with clinical scores >1 were recorded. **B.** As described in (A), mice were injected with CFA-MOG to elicit EAE and were treated with rabbit anti-mouse CD226 pAb (solid bar) or rabbit IgG (open bar). Median incidence time was calculated using Graphpad Prism 5. **C.** As described in (A), mice were injected with CFA-MOG to elicit EAE and were treated with rabbit anti-mouse CD226 pAb (dashed line) or rabbit IgG (solid line). **D.** As described in (A), spinal cords were isolated from mice during the incidence peak (about the 18^th^ d) which treated with CD226 pAb and from mice treated with rabbit IgG. Histopathology of representative lumbar spinal cord sections was analyzed by hematoxylin-and-eosin staining. Boxed areas in the left two panels are shown at ×20 magnification in the right two ones respectively. Bars in the left two panels represent 100μm; bars in the right two panels represent 5μm. **E.** The inflammatory foci (>10 mononuclear cells) infiltrated in the meninges and parenchyma in the spinal cord sections in (D) were counted. Data are representative of three experiments with 12 mice per group (A-C) or one experiment with at least three to five mice per group (D and E). Error bars denote SEM. *P < 0.05, **P < 0.01.

The findings that CD226 ligation could promote IL-10 cytokine secretion led us to further investigate whether CD226 is involved in the development of autoimmune diseases such as EAE, a murine model for human multiple sclerosis, which has been proved to be regulated by Th1/Th17 as well as Tregs [19–21]. After administrated every other day during the first 8 d of EAE elicitation, CD226 pAb efficiently reduced the incidence and postponed the median incidence time of EAE onset (Figure 4A and 4B). In addition, the clinical scores of CD226 pAb treated EAE mice were much lower than that of mice treated with control Ab (Figure 4C), suggesting that administration of CD226 pAb effectively reduced EAE susceptibility and severity. These results were consistent with the findings that anti-CD226 treatment delayed the onset of EAE in mice and inhibited the activation and proliferation of CD4^+^ T cells isolated from MS patients [12, 13].

Histopathological assessment of representative lumbar spinal cord sections demonstrated the potent role of CD226 pAb in reducing the infiltration of inflammatory cells in CNS characterized by less infiltration of parenchymal perivascular mononuclear cells in the meninges and parenchyma which is associated with demyelination compared with that in the control group (Figure 4D and 4E). Therefore, CD226 was highly related to the initiation and maintenance of EAE.

### CD226 pAb promotes splenic CD4^+^IL-10^+^ T cell differentiation in EAE mice

**Figure 5:**
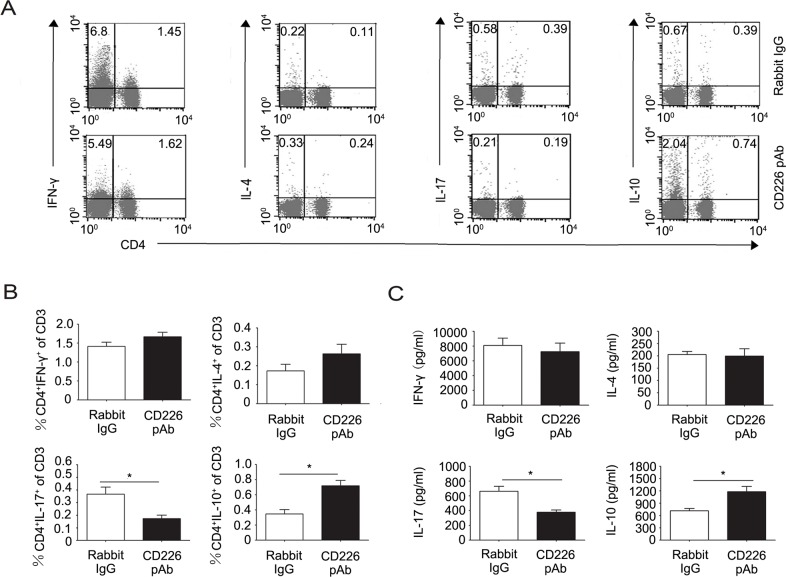
CD226 pAb promotes CD4^+^IL-10^+^ T cell differentiation of splenocytes in EAE mice *in vivo* **A.** EAE was elicited in mice with CFA-MOG injection. Mice were also administrated with rabbit anti-mouse CD226 pAb or rabbit IgG every other day during the first 8 d of EAE elicitation. Splenocytes were isolated from mice with a clinical score of 3 during the incidence peak (about the 18^th^ d) which were treated with rabbit IgG and from mice treated with CD226 pAb for the same periods of time. The frequencies of Th subsets were determined by flow cytometry analysis through detecting surface expression of CD4 *versus* IFN-γ, IL-4, IL-17 and IL-10 intracellular staining on the isolated splenocytes, stimulated with PMA and ionomycin for 4h in the presence of GolgiStop. Cells were stained with LIVE/DEAD Fixable Dead Cell Stain Kit before fixation to allow gating on viable cells. **B.** The frequencies of Th subsets in the total T cells from experiments described in (A) were compared. **C.** MACS sorted CD4^+^ T cells from the isolated mouse splenocytes as described in (A) were activated under Th1, Th2, Th17 or iTreg conditions for 4d. The production of IFN-γ, IL-4, IL-17 and IL-10 in the supernatants was quantified by ELISA. Data are representative of one experiment with at least three to five mice per group. Error bars denote SD (B) or SEM (C). *P < 0.05.

Intrigued by the above observations, we further investigated the mechanisms by which CD226 affects EAE. Considering the *ex vivo* findings that CD226 efficiently inhibited the Th1/Th17 differentiation but promoted iTreg generation in humans, and our finding that CD226 was highly expressed on the surface of mice Th17 and Treg which played important roles in EAE pathologies (data not shown), we speculated that CD226 might be involved in mice EAE through modulating the Th cell subsets and iTreg differentiation. To confirm this hypothesis, splenocytes from the EAE mice administrated with CD226 pAb or control Ab were isolated. Th subsets and associated cytokine expression levels were assessed by intracellular cytokine staining and ELISA respectively. It was found that CD226 pAb treatment strongly decreased the frequency of CD4^+^ IL-17^+^ Th17 while elevated that of CD4^+^ IL-10^+^ T cells with no significant effect on the IFN-γ producing Th1 and IL-4 producing Th2 (Figure 5A and 5B). Meanwhile, CD226 pAb treated EAE mice produced less IL-17 and more IL-10 comparing with that of control mice (Figure 5C).

**Figure 6:**
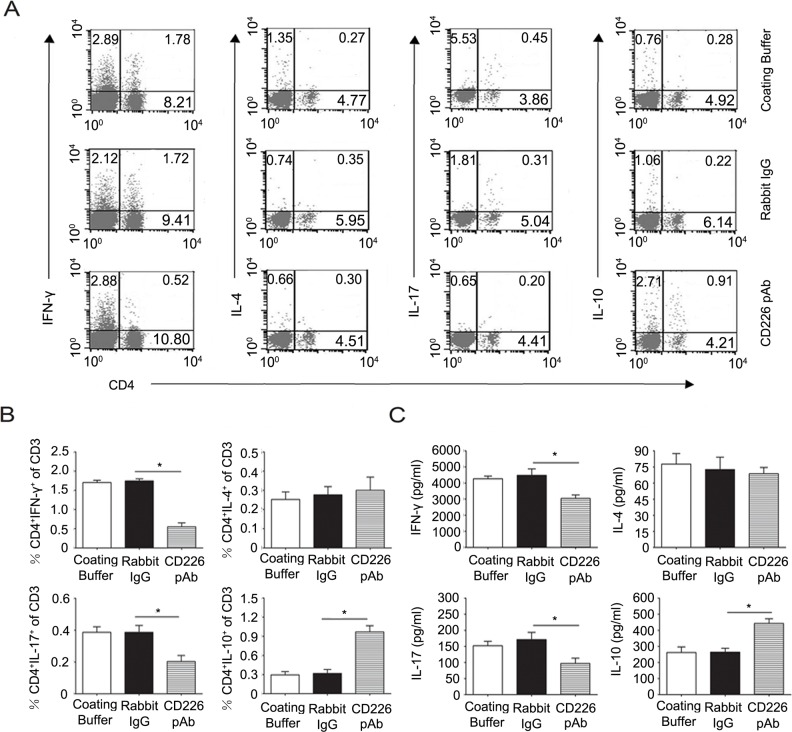
Crosslinked CD226 pAb *in vitro* induces CD4^+^IL-10^+^ T cells differentiation of splenocytes in EAE mice **A.** EAE was elicited in mice with CFA-MOG injection. Splenocytes were isolated from mice with a clinical score of 3 during the incidence peak (about the 18^th^ d) and treated with MOG_35-55_ and plate bound rabbit anti-mouse CD226 pAb, rabbit IgG (isotype control) or only coating buffer (negative control). On day 3, the frequencies of Th subsets were determined by flow cytometry through surface expression of CD4 *versus* IFN-γ, IL-4, IL-17 and IL-10 intracellular staining on the isolated splenocytes, stimulated with PMA and ionomycin for 4h in the presence of GolgiStop. Cells were stained with LIVE/DEAD Fixable Dead Cell Stain Kit before fixation to allow gating on viable cells. **B.** The frequencies of Th subsets in the total T cells from experiments described in (A) were compared. **C.** MACS sorted mouse CD4^+^ T cells from the isolated splenocytes as described in (A) were treated with MOG_35-55_ and plate-bound rabbit anti-mouse CD226 pAb, rabbit IgG or only coating buffer under Th1, Th2, Th17 or iTreg conditions for 3d. The production of IFN-γ, IL-4, IL-17 and IL-10 in the supernatants was quantified by ELISA. Data are representative of one experiment with at least three to five mice per group. Error bars denote SD (B) or SEM (C). *P < 0.05.

To further confirm the *in vivo* results, mice splenocytes were isolated and added into the CD226 pAb pre-coated plate. In line with the *in vivo* data, CD226 pAb crosslinked mice splenocytes showed a lower frequency of CD4^+^ IL-17^+^ but a higher percentage of CD4^+^ IL-10^+^ T cells compared with that of the control ones (Figure 6A and 6B). ELISA results also showed the similar role of CD226 pAb in inhibiting IL-17 and promoting IL-10 secretion (Figure 6C).

### CD226 knockout mice show significantly better outcome of EAE accompanied by higher IL-10 expression levels

**Figure 7:**
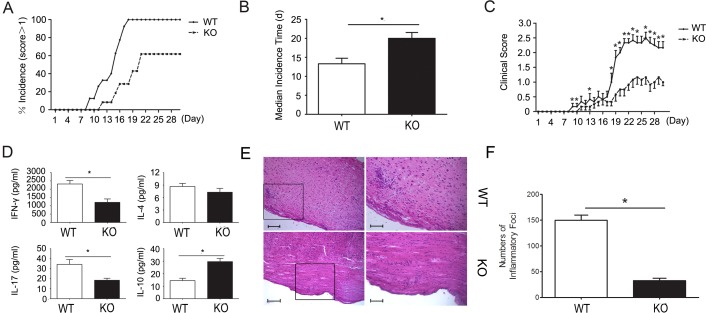
CD226 knockout mice show significantly better outcome of EAE and differentially altered EAE related Th1/Th17 and iTreg associated cytokines **A.** EAE was elicited in CD226^−/−^ (dashed line) and wild type (solid line) mice with CFA-MOG injection. The numbers of mice with clinical scores >1 were recorded. **B.** As described in (A), CD226^−/−^ (solid bar) and wild type (open bar) mice were injected with CFA-MOG to elicit EAE. Median incidence time was calculated using Graphpad Prism 5. **C.** As described in (A), CD226 knockout (dashed line) and wild type (solid line) mice were injected with CFA-MOG to elicit EAE. Clinical scores of EAE were recorded every day thereafter. **D.** As described in (A), CD226 knockout (solid bar) and wild type (open bar) mice were injected with CFA-MOG to elicit EAE. Eye ball venous blood from mice during the incidence peak (about the 18^th^ d) were collected. IFN-γ, IL-4, IL-17, and IL-10 expression levels were determined by ELISA. **E.** As described in (A), spinal cords were isolated from CD226 knockout or WT mice induced with EAE at 18^th^ d to 21^st^ d. Histopathology of representative lumbar spinal cord sections was analyzed by hematoxylin-and-eosin staining. Boxed areas in the left two panels are shown at ×5 magnification in the right two ones respectively. Bars in the left two panels represent 25μm; bars in the right two panels represent 5μm. **F.** The inflammatory foci (>10 mononuclear cells) infiltrated in the spinal cord sections in (E) were counted. Data are representative of three experiments with 12 mice per group (A-D) or one experiment with at least three to five mice per group (E and F). Error bars denote SEM. *P < 0.05.

CD226 knockout mice were used to further assess the effect of CD226 on the induction of EAE. C57BL wild type mice initially showed clinical signs of EAE on day 8 and all became sick on day 11 after EAE elicitation. In contrast, CD226 knockout mice showed clinical signs of EAE on day 11 with 40% remaining healthy till the end of our assay (Figure 7A). Similarly, the median incidence time was also markedly postponed in the CD226 knockout mice compared with their counterparts in the wild type group (Figure 7B). And the mean clinical score of EAE of CD226 knockout mice was significantly decreased since 18 d after the EAE induction (Figure 7C). In addition, CD226 knockout virtually decreased the production of IFN-γ and IL-17 but strongly increased the production of IL-10 with unnoticeable effects on the production of IL-4 (Figure 7D). Furthermore, remarkably reduced infiltration of parenchymal perivascular mononuclear cells was found in the spinal section from CD226 knockout mice as compared with the wild type mice (Figure 7E and 7F). These findings suggested that CD226 has a critical role in mediating MOG_35-55_-induced EAE at least partially *via* inhibiting iTreg and promoting Th1/Th17 associated cytokine production.

## DISCUSSION

In this study, we found that CD226 ligation efficiently inhibited the production of Th1 and Th17 associated cytokines but led to a significant increase of IL-10 expression level which came at least partly from iTregs in PBMC or MLC system. Furthermore, LeoA1 treatment significantly facilitated the iTreg differentiation while suppressing the differentiation of Th1 and Th17. CD226 pAb *in vivo* administration effectively reduced the onset of mice EAE by inhibiting the differentiation of Th1/Th17 but promoting the IL-10-secreting CD4^+^ T cells. Moreover, similar results can be found in CD226 knockout mice. This study therefore reveals the critical role of CD226 in the pathogenesis and treatment of EAE by promoting IL-10-secreting iTregs and inhibiting Th1/Th17 generation.

Previous studies showed that Th1, Th17 and Th9 cells each could transfer EAE with similar severity and overlapping but distinct pathological phenotypes [1], and treatment targeting CD4^+^ T cells differentiation significantly ameliorated EAE [22]. Moreover, suppression of EAE was associated with the decreased frequencies of CD226 expressing CD4^+^ T cells [23], elevated IL-10 expression and increased number of regulatory T cells [24]. Thus, we investigated the effect of CD226 ligation on Th subsets and IL-10 secreting CD4^+^ T cells polarization as well as its contribution to the amelioration of EAE. We claimed that CD226 mAb LeoA1 played a more predominant role in regulating the CD4^+^ Th cells associated cytokines production in MLC system as compared with their counterparts in PBMC system. Furthermore, we think that PBMC is a relatively pure system compared with MLC system. In that system, lymphocytes stay inactivated and always produce a low level of cytokines. Although MLC is a well-known and classical model commonly used in analyzing the HLA antigen compatibility before organ transplantation, the lymphocytes can be activated and proliferate when stimulated by allogeneic antigen on APC and produce a wide range of cytokines in a high level. We found that in both activated T cells and inactivated T cells, CD226 blockage effectively increased the IL-10 production.

Although it has been reported that many immune cells expressed IL-10 including Th and Treg cells, CD8^+^ T cells, B cells, dendritic cells (DCs), macrophages, mast cells, natural killer (NK) cells, eosinophils and neutrophils [25–27], here, we found that CD226 mAb LeoA1 significantly stimulated the CD4^+^IL-10^+^ T cells differentiation. Consistent with these findings, Ester Lozano et al. reported that blockade of T cell Ig and ITIM domain (TIGIT), a transmembrane glycoprotein which expressed on peripheral memory and regulatory CD4^+^ T cells and NK cells and competing with CD226 for binding to the same ligand CD155, decreased the IL-10 expression derived from human CD4^+^ T cells [28].

Inspired by the finding that differentially altered Th subsets associated cytokines were induced by CD226 mAb blockage, we did real-time PCR to confirm its effect on Th and Treg cell differentiation. Considering the purity of RNA isolation, we chose CD3/CD28 system to activate CD4^+^ T cells instead of the MLC, trying to exclude the disturbance of RNA from the APC (Daudi) and other cells (NK, B cells, DC, macrophages) in MLC system. As for the key transcription factors involved in the differentiation of CD4^+^ T cells, we found that CD226 mAb LeoA1 decreased T-bet and RORγt expression and increased Foxp3 expression, skewing cells toward iTreg function while repressing the Th1/Th17 differentiation. In contrast, IL-4 and Gata-3 which are critical in Th2 differentiation were not affected. Contradictorily, it was shown that silencing CD226 expression by lentiviral transduction led to increased Th2 associated cytokines and transcriptional factors [13]. This discrepancy may be resulted from the fact that cells in the neutralization experiments were incubated under Th2-polarizing conditions, thus, expressed lower levels of CD226, inhibiting part of the effects displayed by LeoA1, which has been proved by the differential expression levels of CD226 in human CD4^+^ Th subsets under respective Th-polarizing conditions [13]. Further studies should be focused on the key receptors and transcriptional factors in the differentiation process of Th subsets and iTregs, such as TGF-β receptor II and STAT5, which were closely associated with generation and stability maintenance of iTreg [29], as well as IL-10 receptor I and STAT3, which is critical to IL-10 associated biological functions [30]. Considering that the Foxp3 gene expression was also markedly elevated, and the CD4^+^ T cells were isolated from PBMC then cultured under iTreg differentiation condition with CD3/CD28 Ab rather than generated from thymus, it is reasonable to believe that the upregulated IL-10 secreting CD4^+^ T cells were iTregs instead of nTregs.

Previous studies have shown that anti-CD226 treatment delayed the onset and reduced the severity of Th1-mediated EAE, the mechanism was supposed to be the cell death of Th1 cells or the inhibited migration of inflammatory cells (T cells or macrophages) to CNS[12]. Moreover, loss of the inhibitory molecule TIGIT which competing for the same ligand with CD226, resulted in hyperproliferative T cell responses and increased susceptibility to autoimmunity [31]. Consistently, we found in this study that CD226 pAb treatment effectively reduced the onset and severity of EAE in mice. More importantly, experiments with CD226 knockout mice further proved the protective effects of CD226 ligation, offering solid evidence of the positive effects of CD226 on the pathogenesis of autoimmune diseases manifested here by EAE. Lymphocytes of EAE mice exposed to CD226 pAb further indicated the reciprocally regulated Th1/Th17 and iTreg differentiation by CD226 ligation except the unchanged IFN-γ producing Th1 cell proportion and the IFN-γ production in the *in vivo* experiment. As for this unexpected result, we postulated it might be due to the different action modes of CD226 pAb when administrated *in vivo* or *ex vivo*.

Additionally, we proved that the significantly elevated IL-10 production may contribute to the amelioration of EAE mediated by CD226 pAb treatment or in CD226 knockout mice. Similar to our results, it was reported that immunoregulatory cytokines, including IL-10, inhibited the development of EAE [32]. IL-10 suppresses effector T cell responses and limits inflammation. Furthermore, IL-10-deficient mice develop more severe EAE than wild-type (WT) mice. However, despite the well-established immunoregulatory effects and functions of ectopically expressed IL-10, efficacy of pharmacologically administered IL-10 in EAE might vary with timing and mode of administration, and could even exacerbate disease [33].

In conclusion, we demonstrated that CD226 ligation inhibited the Th1/Th17 differentiation and promoted iTreg differentiation of CD4^+^ T cells. In addition, CD226 plays an important role in maintaining the pathological process and mediating clinical symptoms of EAE *via* stimulating Th1/Th17 differentiation of CD4^+^ T cells. More importantly, we revealed for the first time that promoting IL-10 production and the CD4^+^IL-10^+^ T cells may be a novel mechanism involved in the amelioration of EAE exhibited by CD226 ligation, thus indicating that blocking CD226 molecule may be a novel target for the therapeutic intervention and treatment of MS by enhancing the expression of IL-10 and iTreg differentiation.

## MATERIALS AND METHODS

### Mice

CD226^−/−^ mice in C57BL/6 background were kindly offered by Professor Marco Colonna. C57BL/6 mice of 8 weeks were purchased from Yison BIO (Shanghai) and all animals were maintained under pathogen-free conditions at Experimental Animals Center of Fourth Military Medical University. The Institutional Review Board (IRB) of the Fourth Military Medical University approved the experiments (permit number XJYYLL-2014433) which were performed according to the relevant guidelines and regulations.

### Reagents

Lymphocyte separation medium and FBS were respectively purchased from Millipore and Gibco. Abs to CD226 were generated as previously described [34, 35]. All other reagents were purchased from Sigma-Aldrich (St Louis, MO) except that myelin oligodendrocyte glycoprotein (MOG_35-55_) was purchased from Difco, Detroit, MI. Antibodies for flow cytometry were listed in Table 1.

**Table 1:** Information for the antibodies utilized in flow cytometry assay

Target molecule	Clone	Reactivity	Fluorescence	Corporation
**IL-10**	JES3-9D7	Human	PE	BioLegend
**CD3**	OKT3	Human	APC	BioLegend
**CD4**	OKT4	Human	PerCP/Cy5.5	BioLegend
**CD11c** **CD14** **CD16** **CD19** **CD56** **HLA-DR**	Bu15 HCD14 3G8 HIB19 HCD56 L243	Human Human Human Human Human Human	PerCP/Cy5.5 FITC FITC PerCP/Cy5.5 APC FITC	BioLegend BioLegend BioLegend BioLegend BioLegend BioLegend
**CD4**	RM4-4	Mouse	PerCP/Cy5.5	BioLegend
**IL-4**	11B11	Mouse	PE	BioLegend
**IL-10**	JES5-16E3	Mouse	PE	BioLegend
**IL-17**	FN50	Mouse	PE	BioLegend
**IFN-γ**	XMG1.2	Mouse	PE	BioLegend
**Rat IgG1, κ**	RTK2071	Rat	PE	BioLegend
**Rat IgG2b, κ**	RTK4530	Rat	APC	BioLegend
**Rat IgG2b, κ**	RTK4530	Rat	PerCP/Cy5.5	BioLegend
**Mouse IgG2b, κ**	MPC-11	Mouse	PerCP/Cy5.5	BioLegend
**Mouse IgG2a, κ**	MOPC-173	Mouse	APC	BioLegend

### MLC and PBMC culture systems

Human PBMC (responding cells) were purified by density gradient centrifugation method [36] and cultured with or without x-irradiated (3000 rads) Daudi (stimulating cells) in complete media consisting of RPMI 1640 with 10% FBS, 2 mM L-glutamine, 10 mM Hepes, and 1×pen/strep/glutamine solution (Sigma-Aldrich). The cell concentrations were 1×10^5^ stimulating and 1×10^6^ responding cells, that is 1 ml/well in 24-well U-bottom plate (Costar, Cambridge, MA). The plate was incubated at 37°C in the presence of 5% CO_2_ for the indicated periods of time with the supernatants or cells to be tested.

### Measurement of cytokine production in culture supernatants and serum

Culture supernatants were harvested at the indicated time point, serum of mice eye ball venous blood was also collected. IFN-γ, TNF-α, IL-2, IL-12, IL-4, IL-17, IL-23 and IL-10 expression levels were measured using the respective ELISA kits (R&D Systems; eBioscience) following the manufacturer's instructions. Absorbance was measured at 450 nm using a Benchmark microplate reader (Bio-Rad).

### Intracellular cytokine staining

Before detection of intracellular cytokine production, human PBMC or isolated mice splenocytes were restimulated with PMA (50 ng/ml) plus ionomycin (500 ng/ml) for 4h in the presence of Brefeldin A (GolgiStop; BD Biosciences). Staining with LIVE/DEAD Fixable Dead Cell Stain Kit (Molecular Probes) was performed before fixation to allow gating on viable cells. Cells were then blocked for 15 mins and stained with antibodies targeting specific surface markers for different lymphocytes (APC anti-CD3, PerCP/Cy5.5 anti-CD4/ PerCP/Cy5.5 anti-CD19/ FITC anti-CD16, APC anti-CD56/ FITC anti-HLA-DR, PerCP/Cy5.5 anti-CD11c/ FITC anti-CD14) at 4°C for 30 mins. After fixation and permeabilization with Fixation and Permeabilization solution (BD Biosciences) for 20 mins, cells were stained with PE anti-IFN-γ, PE anti-IL-4, PE anti-IL-17, PE anti-IL-10 or PE Rat IgG1, κ (BioLegend) 4°C for 30 mins respectively and then submitted to flow cytometry analysis (FACS Calibur; BD Biosciences).

### *In vitro* T cell differentiation assays

Human CD4^+^ T cells and mice CD4^+^ T cells were purified using negative selection (CD4^+^ T Cell Isolation Kit, human; CD4^+^ T cell Isolation kit, mouse; Miltenyi Biotec, Auburn, CA) respectively. Purified human and mice CD4^+^ T cells were stimulated in the presence of anti-CD3 (5 μg/ml, UCHT1; 1 μg/ml, 145-2C11) and anti-CD28 (1 μg/ml, CD28.2; 1 μg/ml, 37.51) (BD Bio-sciences) for 3d under the following conditions—For human CD4^+^ T cells: Th1: rhIL-12 (10 ng/ml; Peprotech, Rocky Hill, NJ); Th2: rhIL-4 (25 ng/ml; Peprotech); Th17: TGF-β (5 ng/ml; Peprotech), IL-1β, IL-21 and IL-23 (all 25 ng/ml; Peprotech); Treg: 1 ng/ml rhTGF-β and 2 ng/ml rhIL-2 (Peprotech). Recombinant human IL-2 was bought through ACRO Biosystems and was used at 10 U/ml, except under Treg (2 ng/ml) and Th17 conditions. For mice CD4^+^ T cells: Th1: 40 ng/ml rIL-12 (Peprotech) and 40 μg/ml anti-IL-4 (R & D); Th2: 40 ng/ml rIL-4 (Peprotech) and 10 μg/ml anti-IFN-γ (R & D); Th17: 1 ng/ml rTGF-β and 40 ng/ml rIL-6 (both Peprotech), 40 μg/ml anti-IL-4 and 10 μg/ml anti-IFN-γ (both R & D); Treg: 1 ng/ml rTGF-β and 2 ng/ml rIL-2 (both Peprotech).

### Real-time quantitative RT-PCR

Purified human CD4^+^ T cells were cultured in Th1, Th2, Th17 or iTreg conditions in the presence of anti-CD3 and anti-CD28 for 3d before total RNA was isolated with TRIZOL (Invitrogen, Carlsbad, CA). cDNA was synthesized with PrimeScript™ RT Master Mix (Takara). Real-time quantitative polymerase chain reaction (PCR) was performed using 7500 Fast Real-Time PCR System (Applied Biosystems, Forest City, CA) according to the manufacturer's instructions with SYBR^®^ *Premix Ex Taq*™ II (Takara). The gene-specific primer sequences were displayed as in Supplemental Figure 2. Relative expression levels of genes were normalized within each sample to the expression of GAPDH. Expression of genes was determined relative to Hprt by the ΔΔCT method.

### EAE induction, evaluation and administration with CD226 pAb

EAE was induced in female C57BL/6 mice. 24 Mice were injected subcutaneously with an emulsion containing 200μg of MOG35-55 (MEVGWYRSPFSRVVHLYRNGK) and complete freund's adjuvant (CFA) supplemented with 200μg of H37RA in the posterior right and left flank; Mice were also injected with 75 ng and 200 ng PTx intraperitoneally on day 0 and 2 relative to immunization. One week later, all mice were similarly injected at 2 sites on the right and left flank anterior of the initial injection sites (2×MOG_35-55_+CFA). The mice were assessed for signs of EAE according to the following scale: 0, normal; 1, limp tail or mild hind-limb weakness; 2, moderate hind-limb weakness or mild ataxia; 3, moderately severe hind-limb weakness; 4, severe hind-limb weakness or mild fore-limb weakness or moderate ataxia; 5, paraplegia with no more than moderate fore-limb weakness; and 6, paraplegia with severe fore-limb weakness or severe ataxia or moribund condition. Rabbit anti-mouse CD226 pAb or rabbit IgG was injected i.p. at a dose of 5 mg/kg every other day from day 0 to day 8.

### Histopathology analysis

Mice induced with EAE at 18^th^ d to 21^st^ d were sacrificed. Spinal cords were harvested and fixed in 10% neutral-buffered formalin and processed routinely for paraffin embedment. Slides were stained with hematoxylin-and-eosin and the images were captured by Nikon Eclipse E200 microscopy (Melville, NY). Histological lesion was evaluated by counting the inflammatory foci (>10 mononuclear cells) in the meninges and parenchyma in a blinded fashion in that the pathologist was unaware of the clinical status.

### Statistical analysis

Real time RT-PCR, ELISA, and survival analysis data were analyzed with GraphPad Prism version 5.0 (GraphPad Software, San Diego, CA). Flow cytometry data were analyzed with FlowJo software (TreeStar, Ashland, OR). Data representing the means ± standard deviation (SD) or means ± standard error (SE) of the results were compared by either the two-tailed Student t test (two groups) or ANOVA (multiple groups), with a posthoc Tukey test to determine significance. All analyses were performed using GraphPad Prism version 5.0. A *p* value ≤ 0.05 was considered statistically significant.

## SUPPLEMENTARY MATERIAL FIGURES


