# Supplementary material for: CD226 ligation protects against EAE by promoting IL-10 expression via regulation of CD4+ T cell differentiation
Source: Oncotarget. 2016 Mar 1;7(15):19251–64. doi: 10.18632/oncotarget.7834 (PMC4991380; doi:10.18632/oncotarget.7834)
Supplement: Supplementary file 1 [file oncotarget-07-19251-s001.pdf]

CD226 ligation protects against EAE by promoting IL-10 expression *via* regulation of CD4$^+$ T cells differentiation

Supplementary Material

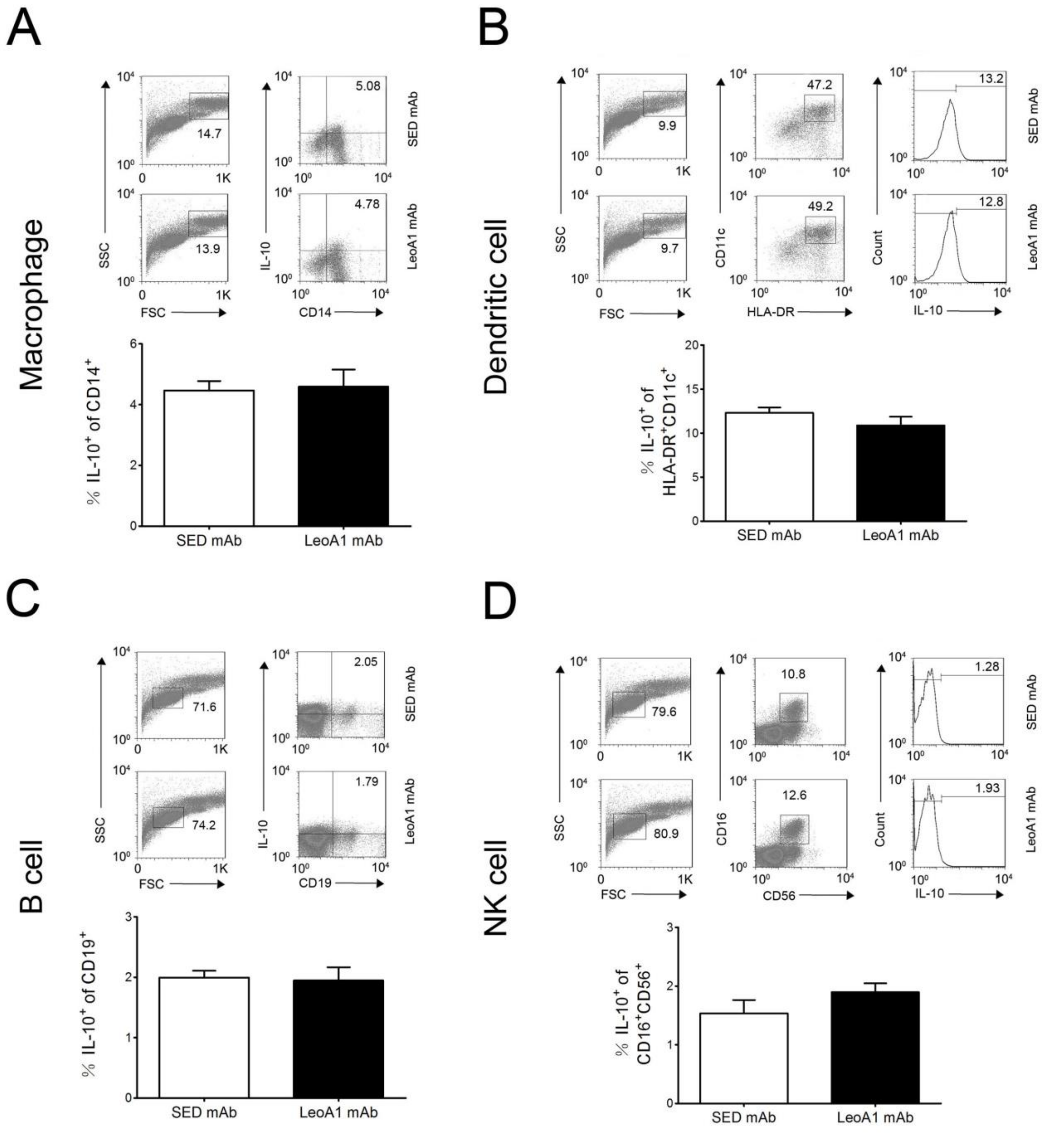

Supplemental Figure 1: CD226 mAb LeoA1 showed no effect on IL-10$^+$ proportion of macrophages, dendritic cells, natural killer cells and B cells in PBMC. Human PBMC were

cultured with LeoA1 or SED mAb for 24h under MLC system and submitted to flow cytometry analysis by gating on specific surface markers of macrophages (A), dendritic cells (B), B cells (C) and natural killer cells (D) followed by IL-10 and isotype control antibody intracellular staining on cells, stimulated with PMA and ionomycin for 4h in the presence of GolgiStop. Cells were stained with LIVE/DEAD Fixable Dead Cell Stain Kit before fixation to allow gating on viable cells. The frequencies of IL-10^{+} cells in each population were compared. Data are representative of at least three independent experiments. Error bars denote SD.

Target gene	NM number	Primer sequence (5'-3')
IL-4	NM_000589.3	TTGAACAGCCTCACAGAGCAGA<br>GTTGTGTTCTTGGAGGCAGCA
IL-10	NM_000572.2	GAGATGCCTTCAGCAGAGTGAAGA<br>AAGGCTTGGCAACCCAGGTA
IL-17A	NM_002190.2	GCAGGCACAAACTCATCCAT<br>CAGGCTCAGCAGCAGTAGCA
IFN-γ	NM_000619.2	CTTTAAAGATGACCAGAGCATCCAA<br>GGCGACAGTTCAGCCATCAC
RORC	NM_001001523.1	GCCAGAATGACCAGATTGTGCTT<br>AAGGCACTTAGGGAGTGGGAGA
GATA-3	NM_001002295.1	AGGACGAGAA AGAGTGCC<br>GAAGAGTCCGGAGCTGTAC
Foxp3	NM_001114377.1	CTGACCAAGGCTTCATCTGTG<br>ACTCTGGGAATGTGCTGTTTC
T-bet	NM_013351.1	TGGGTGCAGTGTGGAAAGGC<br>ACTGGAGCACAATCATCTGGG
GAPDH	NM_001256799.2	TCACCACCATGGAGAAGGC<br>GCTAAGCAGTTGGTGGTGCA

Supplemental Figure 2: Primer sequences used in the real-time PCR. Primer sequences and associated NM number in Pubmed of the target molecules used in the real-time PCR were listed.
